# Genome Sequence of the Anterograde-Spread-Defective Herpes Simplex Virus 1 Strain MacIntyre

**DOI:** 10.1128/genomeA.01161-14

**Published:** 2014-11-13

**Authors:** Moriah L. Szpara, Yolanda R. Tafuri, Lance Parsons, Jacob T. Shreve, Esteban A. Engel, L. W. Enquist

**Affiliations:** a Department of Molecular Biology, the Princeton Neuroscience Institute, Princeton University, Princeton, New Jersey, USA; b Lewis-Sigler Institute for Integrative Genomics, Princeton University, Princeton, New Jersey, USA; c Department of Biochemistry and Molecular Biology, Pennsylvania State University, University Park, Pennsylvania, USA

## Abstract

We used paired-end Illumina deep sequencing and *de novo* assembly to determine the genome sequence of herpes simplex virus 1 (HSV-1) strain MacIntyre (aka McIntyre). The MacIntyre strain originated from the brain of a patient with lethal HSV encephalitis and has a unique limitation in its neuronal spread, moving solely in the retrograde direction.

## GENOME ANNOUNCEMENT

We used Illumina deep sequencing and *de novo* assembly to determine the genome sequence of herpes simplex virus 1 (HSV-1) strain MacIntyre (also spelled MacIntyre B, McIntyre, and McIntyre B). The MacIntyre strain originated from the brain of a patient with lethal HSV encephalitis (1). After isolation, MacIntyre was passaged through multiple cell types and species (1, 2). Dowdle (2, 3) deposited this strain at the ATCC (VR-539), from which we received our stock. Later studies revealed a severe defect in the anterograde spread of HSV-1 MacIntyre in the central nervous systems in rat, mouse, and primate models (4–12). After replication in the nucleus, HSV-1 MacIntyre egresses and infects only neurons that are presynaptic to the infected neuron. This is in contrast to wild-type HSV-1, which egresses and infects neurons both pre- and postsynaptic to the infected one.

We isolated viral nucleocapsid DNA from HSV-1 MacIntyre-infected Vero cells, using published methods (13–15). This was performed according to the manufacturer’s protocols (Illumina TruSeq DNA) to produce a bar-coded library of 500-bp fragments and to obtain 100-bp paired-end sequence reads (Illumina HiSeq). A series of quality control filters removed the sequences resulting from Illumina primers and adaptors, contaminating Vero cell DNA, and low-quality terminal bases (16, 17). We then generated eight SSAKE *de novo* assemblies using varied parameters, and we combined these into a draft genome using Celera and GapFiller (18–21). We transferred annotations from the HSV reference genome (strain 17; GenBank accession no. JN555585) to the MacIntyre strain based on sequence homology (22, 23). Three genes, UL46, US7, and US9, contained new stop codon positions due to homopolymer-based frameshifts or single nucleotide variations. Two genes (RL1 and RS1) were undetermined due to gaps in the assembly. As in prior studies (17, 24–29), we found that a majority of HSV MacIntyre proteins have coding variations compared to those of the HSV-1 reference strain 17. We anticipate that the HSV-1 MacIntyre genome contains bystander variations and one or more mutations that directly affect its limited-spread phenotype.

A frequent comparator for HSV-1 is the distantly related swine alphaherpesvirus pseudorabies virus (PRV) (30, 31). HSV-1 MacIntyre resembles the PRV vaccine strain Bartha in terms of its defect in anterograde spread, extensive passage history, and attenuated virulence (32, 33). We recently sequenced the full genome of PRV strain Bartha, allowing us to explore how these two viruses converged on the same phenotype of defective spread in neurons (16). The anterograde spread defect of PRV-Bartha results from loss of three proteins, gE (US6), gI (US7), and US9. The loss of US9 alone strongly affects sorting into neuronal axons (34–37). Our sequence data reveal that HSV-1 MacIntyre contains a single nucleotide polymorphism in US9, which creates a premature stop codon (C172T or R58Stop). We have confirmed this by PCR and Western blot analysis (data not shown). Curiously, an identical US9 mutation was previously described in two additional unrelated HSV-1 strains (38, 39). Further characterization of this and other differences in HSV-1 MacIntyre is under way with an aim of illuminating the mechanisms of neuronal sorting and egress for HSV-1.

### Nucleotide sequence accession number.

The HSV-1 MacIntyre strain genome sequence has been deposited at GenBank under the accession no. KM222720.
